# The chief complaint driven medical history: implications for medical education

**DOI:** 10.5116/ijme.5907.74d8

**Published:** 2017-05-30

**Authors:** Richard Nierenberg

**Affiliations:** 1 Emergency Medicine, Hackensack University Medical Center Site, Saint George University School of Medicine, Grenada West Indies

**Keywords:** The chief complaint, driven medical history, medical education

## Introduction

It is the purpose of this paper to present an approach to teaching a concise, focused method to obtain a medical history from patients in the Emergency Department. Diagnostic reasoning starts with the medical history. Nowhere is obtaining an accurate history more important than in the Emergency Department.  Here the demand for a quick focused and effective assessment and presentation prompts some to propose that medical students be able to accurately present a case in as little as “three minutes”.^1^

The key is for the student is to learn to determine what pertinent information is. There exists a robust history of inquiry into how expertise is developed in clinical reasoning.^2^ Experienced clinicians work through the early development of diagnostic hypotheses which they then use to account for clinical findings. This problem solving method was initially referred to as “hypothetico-deductive” reasoning.^3^

Rather than a general reasoning skill, it was soon found that clinical reasoning was dependent on a specific knowledge domain, that diagnostic accuracy depends more on mastery of specific medical content than it does on general diagnostic strategy.^4^^,^^5^

Case relevant recall differs between expert and novice clinicians.^6^   Experts have more accurate initial diagnoses. However, they do not recall more total information about a presentation. They recall more relevant information. Experts make better selective use of data, choosing relevant over relevant to retain, retrieve and apply.    

To learn how to determine relevance, the clinician must learn to apply certain patterns to organize patient information and relate it to a structured knowledge base. Students learn to place data and knowledge into larger units of meaning and connect the units of meaning together to form higher structures of meaning.^7^

One explanation for how novices learn to organize data to separate relevant from non-relevant pieces of information uses the idea of “semantic qualifiers”, which are oppositional, contrasting or dichotomous relationships.^7^   A clinical complaint can be described along a number of different dichotomous axes.  These dichotomies can be used to compare, contrast, and draw distinctions between diagnostic possibilities.

Consider the chief complaint of “chest pain”, one of the most frequent reasons for visits to an emergency department. The chest pain can be characterized as either acute or chronic, sharp or dull, constant or intermittent. It can be associated with dyspnea or not, and occur in the context of multiple risk factors or not. These axes are of key importance, both to represent the problem adequately, and in the formation of clinical meaning for the elements of the history as they pertain to the differential diagnosis.

The traditionally taught method^8^ of obtaining a medical history acquires elements of the history in sequential separate categories.  What has been called the “History of the Present Illness” starts with describing the ‘chief complaint’. This History of the Present Illness is then followed in order by the Past Medical History, Family History, Social History, and Review of Systems. Only after gathering each bit of history in this separate manner are students asked to integrate the acquired data into a whole.

Experts in the emergency setting do not gather or present information in that manner. Clinicians in the emergency department use a combination of simultaneous problem solving and hypothesis generating and testing. As early as first hearing the chief complaint, the expert clinician begins immediately to head toward a diagnosis and evaluates each of several competing diagnostic hypotheses. For each diagnostic possibility, the provider specifically seeks and selects elements from other areas of the history, namely from the ‘past medical history’, ‘family history’, ‘social history’ and ‘review of systems’, which may lead one toward, or away, from each possible diagnosis.  These form the relevant or pertinent positives and negatives. Knowledge of what is pertinent separates the expert from the novice.

Each diagnosis in the differential generates for the experienced clinician questions than can help either rule out or make that diagnosis more or less likely. Using generally dichotomous questions to separate among possibilities allows for better prioritization of steps for further diagnosis and treatment.

Teaching students to use specific questions to point toward or away from a diagnostic possibility, we are asking them to find, use and learn specific differentiating features to compare and contrast potential diagnoses for a given complaint.  Through the process of composing questions to next ask, students learn to discover and articulate which elements of the illness, are most important. They learn which questions have the highest yield in separating one possible diagnosis from another.

### The Example of Abdominal Pain

The causes of abdominal pain differ depending on where exactly in the abdomen pain is located, so the first question to ask any patient who presents with abdominal pain is “Where exactly in the abdomen is this pain located?” In the case of mid-epigastric pain, for example, there are a number of clearly separate causes for such pain.^9^ Developing a differential diagnosis of the causes for this particular patient’s mid-epigastric pain is useful in determining priorities for laboratory and imaging studies.

A chief complaint driven history using the example of mid-epigastric pain would make use of specific dichotomous axes, to ask specifically targeted questions meant to separate, quickly and directly, among the more likely diagnostic entities. For example, pancreatitis is frequently caused by alcohol use, so a very early question in the evaluation of mid-epigastric pain would be whether the patient had a history of alcohol use. As mid-epigastric pain can be cardiac, one would ask presence or absence of dyspnea. The answers to these specific questions will help the clinician prioritize more or less likely causes for the patient’s pain.  Additionally, using specific questions will help the learning clinician to develop a knowledge and experience of those aspects of the history which help define a particular disorder, and help the student develop an “illness script” to help recognize the disorder when seen the next time.

In considering several diagnostic possibilities, the clinician may choose from a few high yield questions which will help make one diagnosis more likely than another. These questions are based along the lines of dichotomous and differentiating units of meaning. The intention of teaching this method of reasoning is that the novice clinician will soon acquire an arsenal of specific questions helping to differentiate among competing diagnoses and move more quickly and effectively toward the more likely diagnoses.

## Conclusions

The current proposal has some implications for medical student education in the emergency department, but I believe this way of teaching is important in other clinical situations as well. The capacity to perform a clear, concise, accurate and focused history and to present the data will be of use in any clinical setting.

It is proposed here that the traditional way in which the medical history is taught must be refined to develop the history taking which is most effective in the busy and diagnostically ambiguous setting of the emergency department, but also in any clinical situation.

After reviewing a selection of the cognitive science of medical education, an approach is proposed for more focused practice, to obtain the medical history in a fashion driven by the chief complaint and guided by a differential diagnosis. Certain specific questions, chosen to separate diagnostic possibilities are chosen to arrive at the most relevant diagnoses quickly.

Future work could focus on empiric demonstrations of the effectiveness of this approach in streamlined acquisition of patient care information both in clinical settings, and those ever present evaluative situations.

### Conflict of Interest

The author declares that they have no conflict of interest.
